
JOURNAL INFORMATION
==============================
NLM Title Abbreviation: J Comp Med Vet Arch
NLM Journal ID: 17840220R

The Journal of Comparative Medicine and Veterinary Archives

Publisher: Biomedical Journal Digitization Project

ARTICLE INFORMATION
==============================
PMCID: PMC9346805
PMID: 36393671
Article version: 1
Subjects: Notes

Notes

Print publication date: 1900 Sep
Volume: 21
Issue: 9
First page: 582
Last page: 582
License: This work is free of known copyright restrictions. For more information, please see PMC Back Issue Digitization.
License URL: https://creativecommons.org/publicdomain/mark/1.0/

==============================

### NOTES.

Mr. Lyman E. Albee, of Fitchburg, Mass., died recently from glanders, contracted in treating a glandered horse. A son is seriously ill from the same disease. The horse was a condemned one, but the owner persisted in treating him.

*A Veterinary Lawyer.*—Mr. Crimsonbeak: They call him a one-horse lawyer.

Mr. Yeast: How did he get that name do you suppose?

"Because he's such a fine charger, I imagine."

Merck & Co., the well-known firm of special preparations of medicinal value, have entered the field of veterinary medicine, and products that are offered are preceded by specially prepared literature, giving full description, properties and actions of the drugs.

City Veterinarian George S. White, of Nashville, Tenn., reports but little meat unfit for food, some milk not up to standard, but many dairies in a filthy condition, all of which is in line for better conditions in these needful sanitary directions.

It is evident that Germany recognizes the value of the veterinary sciences, and is willing to pay for developing them. This is shown by the facts that Hanover has recently erected a new set of buildings for the veterinary school at a cost of nearly 1,000,000 marks; that Saxony has provided 600,000 marks for additions to the hospitals and hygienic department of the veterinary school at Dresden, and at Leipzig the University has set aside 400,000 marks for improving its Veterinary Department. The Agricultural Department of Saxony estimates that the cattle of that State were injured by foot-and-mouth disease during the year 1899 to the extent of 20,000,000 marks. This does not include the losses caused by the ravages of this disease among sheep, goats, and swine.

---

**FOR SALE. BARGAIN.**

A rare chance for a Veterinarian to secure a good established practice of twelve years in a town of 2500 population in Western New York. Fine farming locality, no competition nearer than nine miles. Also, in connection with above practice, a first-class horse-shoeing trade, averaging $15.00 per day year round. Good reasons for selling; terms easy. Do not write unless you mean business and have $500, then address

JOURNAL OFFICE.
